# Diffusion-Controlled Reactions: An Overview

**DOI:** 10.3390/molecules28227570

**Published:** 2023-11-13

**Authors:** Denis S. Grebenkov

**Affiliations:** Laboratoire de Physique de la Matière Condensée, CNRS—Ecole Polytechnique, Institut Polytechnique de Paris, 91120 Palaiseau, France; denis.grebenkov@polytechnique.edu

**Keywords:** diffusion, surface reaction, heterogeneous catalysis, confinement, geometric complexity, biochemistry, reversible reactions, encounter-based approach, Brownian motion, encounter-dependent reactivity

## Abstract

We review the milestones in the century-long development of the theory of diffusion-controlled reactions. Starting from the seminal work by von Smoluchowski, who recognized the importance of diffusion in chemical reactions, we discuss perfect and imperfect surface reactions, their microscopic origins, and the underlying mathematical framework. Single-molecule reaction schemes, anomalous bulk diffusions, reversible binding/unbinding kinetics, and many other extensions are presented. An alternative encounter-based approach to diffusion-controlled reactions is introduced, with emphasis on its advantages and potential applications. Some open problems and future perspectives are outlined.

## 1. Introduction

The nineteenth century was marked by impressive advances in the theory of chemical reactions, even though the existence of atoms and molecules, the (quantum) origins of chemical bonds, and many other fundamental aspects remained to be clarified. Understanding of stoichiometric relations between reactants and the development of a mathematical theory of ordinary differential equations (ODEs) provided a powerful tool to describe the kinetics of very sophisticated reactions. On a basic level, stoichiometric relations allow one to calculate the right proportions of ingredients and the masses of produced reactants at the end. Moreover, they determine the form of the ODEs that govern the time evolution of concentrations of the reactants. For instance, upon disintegration of a substance *A*, its concentration [A] obeys the simplest ODE,

(1) $$\frac{d[A]}{dt}=-k_{A}\left[A\right],$$

where $k_{A}$ is the disintegration rate; here, the change with time of the concentration on the left-hand side is proportional to the remaining concentration on the right-hand side. The solution of this equation, $\left[A\right]\left(t\right)={\left[A\right]}_{0}\exp\left(-k_{A}t\right)$, shows an exponential decay of the concentration from the initial level ${\left[A\right]}_{0}$. The simplicity of this solution is caused by the *linearity* of the equation. For instance, the dynamics of a bimolecular synthesis reaction,

(2) A+B→AB,

is much more sophisticated as being described by *nonlinear* differential equations such as

(3) $$\frac{d[A]}{dt}=-k_{AB}\left[A\right]\left[B\right],$$

in which the rate of decrease in the concentration [A] is proportional to the product of the concentrations of both substances, i.e., to the likelihood of meetings between reactants *A* and *B*. More generally, stoichiometric relations, which determine how many copies of each reactant molecule are involved in a chemical reaction, set the powers of the involved concentrations. The nonlinearity of ODEs describing chemical kinetics presents one of the major mathematical challenges for their analysis, but also the origin of many peculiar features (e.g., non-existence or non-uniqueness of the solution, a finite time to the extinction of some reactants, etc.). These features and their implications in chemistry and biology have been thoroughly investigated in the twentieth century [1,2].

The above description totally ignores the spatial aspects of chemical reactions, as if the concentrations of reactants were homogeneous in space at any time. This is known as the *well-mixed assumption*, where the reactants are assumed to be well mixed so that the reaction occurs in different points in space in the same way. However, there are numerous situations in which the spatial aspects are critically important. For instance, many biochemical reactions in living cells involve proteins and macromolecules that are produced in one spatial location but have to diffuse to another location to find their reaction partners (e.g., receptors, enzymes, or specific sites on DNA chains). Even for small particles such as oxygen molecules, ions, and metabolites, there is generally a gradient of concentration between their “source” and “sink” that drives their directional transport in space. Moreover, even if the concentrations [A] and [B] are macroscopically homogeneous but low, the single molecules *A* and *B* have to meet each other to form an aggregate AB according to the reaction (2), and this transport step takes time and can be the limiting factor in the overall reaction rate. The crucial role of diffusion was put forward by M. von Smoluchowski, who formulated in 1917 the first mathematical description of coagulation dynamics [3], which later became the cornerstone of the theory of diffusion-controlled reactions in a much broader context [4,5,6,7,8,9,10]. Examples of diffusion-controlled reactions include coagulation dynamics [3,11], most catalysis and enzymatic reactions [12,13] and ligand–protein associations [14,15,16], geminate recombination of radicals and ions [17,18], reactions in micellar and vesicular systems [19], spin relaxation on magnetic impurities [20,21], diffusive search by a transcription factor protein for a specific binding site on a DNA molecule [22,23,24], self-propulsion of active colloids [25,26,27], and oxygen capture in the lungs [28,29,30]. Note that such reactions bear other names such as diffusion-limited, diffusion-mediated, diffusion-assisted, or diffusion-influenced reactions. In the past, these names were sometimes used to distinguish the role of diffusion, e.g., whether the reaction occurs instantly upon the first encounter of the reactants, or after an additional chemical kinetics step. We do not make such distinctions and understand diffusion-controlled reactions in a broad sense as reactions in which diffusion is relevant.

In this concise review, we focus on the spatial aspect of chemical reactions. In Section 2, we describe a chemical transformation on a catalytic surface and emphasize the role of diffusion and the consequent spatial dependence of the concentration (e.g., the formation of a depletion zone). Section 3 presents a more realistic setting of imperfect surface reactions, which combine diffusion in the bulk and chemical kinetics on the surface. In Section 4, we briefly give an overview of various extensions such as anomalous diffusion, reversible binding/unbinding reactions, reactions in dynamically heterogeneous media, etc. Section 5 describes an alternative approach to diffusion-controlled reactions based on the statistics of encounters between the reactant and the catalytic surface, while Section 6 concludes the review.

## 2. The Role of Diffusive Transport

For the sake of clarity, we focus on heterogeneous catalysis, where a reactant *A* can be transformed into a product *B* in the presence of an immobile catalyst *C*:

(4) A+C→B+C.

**Figure 1:**
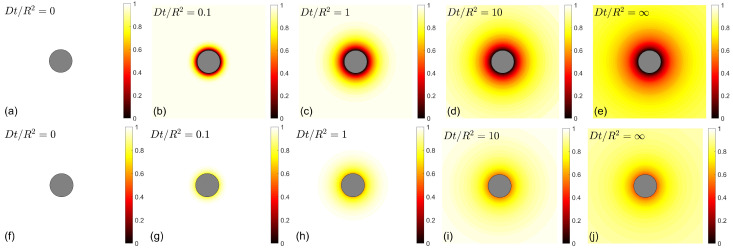
(**Top row**) Rescaled concentration $\left[A\right]\left(x,t\right)/{\left[A\right]}_{0}=1-\frac{R}{\left|x\right|}\mathrm{erfc}\left(\left(|x|-R\right)/\sqrt{4Dt}\right)$ of reactant *A* near a perfectly reactive catalytic sphere of radius *R* (in gray) at different time instances (here erfc(z) is the complementary error function) [3]. (**a**) Homogeneous concentration at t=0; (**b**) Formation of a thin depletion zone in a short time $Dt/R^{2}=0.1$; (**c**,**d**) Progressive growth of the depletion zone at longer times $Dt/R^{2}=1$ and $Dt/R^{2}=10$; (**e**) Approach to a steady-state concentration $\left[A\right]\left(x,\infty \right)/{\left[A\right]}_{0}=1-R/\left|x\right|$ as t→∞. (**Bottom row**) Rescaled concentration $\left[A\right]\left(x,t\right)/{\left[A\right]}_{0}=1-\frac{R-R_{\kappa }}{\left|x\right|}\left\{\mathrm{erfc}\left(\frac{|x|-R}{\sqrt{4Dt}}\right)+e^{Dt/R_{\kappa }^{2}+\left(|x|-R\right)/R_{\kappa }}\mathrm{erfc}\left(\frac{|x|-R}{\sqrt{4Dt}}+\frac{\sqrt{Dt}}{R_{\kappa }}\right)\right\}$ of reactant *A* near a partially reactive catalytic sphere of radius *R* [31], with reactivity κR/D=1 and $R_{\kappa }=R/\left(1+\kappa R/D\right)$), at the same time instants: t=0 (**f**), $Dt/R^{2}=0.1$ (**g**), $Dt/R^{2}=1$ (**h**), $Dt/R^{2}=10$ (**i**), and t=∞ (**j**).

If the catalytic germs were uniformly dispersed in a chemical reactor, one could still rely on Equation (1). However, in many practical situations, catalytic germs have specific locations, most often on a *surface* of a porous medium, so that the reactant *A* should first reach this spatial location. As reactant *A* nears the catalytic germs it has a higher chance to react on them and, thus, to be transformed to *B*, so the concentration [A] becomes space-dependent. In particular, a depletion zone, with a low concentration of *A*, is formed near the catalytic surface (Figure 1, top row). This is a direct consequence of the transport step, which can be described, as in the case of coagulation dynamics, by the diffusion equation (also called the Smoluchowski equation or heat equation),

(5) $$\frac{\partial [A]}{\partial t}=D\Delta \left[A\right],$$

where $\Delta =\partial^{2}/\partial x^{2}+\partial^{2}/\partial y^{2}+\partial^{2}/\partial z^{2}$ is the Laplace operator, and *D* is the diffusion coefficient of reactant *A* in a liquid. In analogy to Equation (1), this equation describes the time evolution of the concentration [A](x,t) in each spatial point x due to diffusive displacements of the reactant *A* in the bulk. In turn, the reaction itself, which occurs on the catalytic surface, *C*, is implemented via a *boundary condition* on that surface. If *A* is transformed into *B* immediately upon the first encounter with the catalyst *C* (so-called perfect reactions), the concentration [A] is set to zero on *C*. This so-called Dirichlet boundary condition was first imposed by von Smoluchowski and still remains the most well-studied and frequently used boundary condition. Its effect is illustrated in Figure 1 (top row) by the dark color near the surface of a spherical catalyst. Note that the overall reaction rate is determined by the diffusive flux of reactant *A* onto the catalytic surface *C*:

(6) $$J\left(t\right)=\int_{C}dx\left(-D\frac{\partial [A](x,t)}{\partial n}\right),$$

where $\partial /\partial_{n}=\left(\vec{n}\cdot \nabla \right)$ is the normal derivative along the normal direction $\vec{n}$ to the surface.

The inclusion of space dependence into the theory of chemical kinetics led to many fundamental changes. As the reaction does not occur homogeneously in space anymore, there are two consecutive steps: the diffusion step (transport towards the catalytic surface described by the diffusion equation) and the reaction step (chemical transformation from *A* to *B* on the catalytic surface, described by the boundary condition). The dependence of these two steps on the shape of the catalytic surface introduces a new *geometric* dimension to the theory [32,33,34]. How efficient are catalytic surfaces of different shapes? Since reaction occurs on the catalytic surface, can *irregularly shaped* catalysts speed up the overall production due to their higher surface area? Can one optimize the shape to increase the production? Have the large reactive surfaces of exchange organs such as lungs and placentas been optimized by evolution for more efficient oxygen capture? These and many other questions have been intensively studied since the 1980s (see [30,35,36,37,38,39,40,41,42] and references therein).

## 3. Imperfect Surface Reactions

Despite their “popularity” among theoreticians, perfect surface reactions ignore intrinsic chemical kinetics during the reaction step and, therefore, may lead to paradoxical predictions. For instance, the overall reaction rate on a perfectly reactive sphere of radius *R*,

(7) $$J\left(t\right)=4\pi RD{\left[A\right]}_{0}\left(1+\frac{R}{\sqrt{\pi Dt}}\right),$$

found by von Smoluchowski [3] is infinitely large at the very first time instant (as t→0). This divergence is caused by the molecules in the immediate vicinity of the catalyst that react instantly. As a consequence, if one searches to maximize the overall production by distributing a given amount of a catalytic material, the optimal solution consists in dispersing this material into a “dust”, i.e., a uniform arrangement of tiny catalytic germs. Moreover, if the subdivision of this material into smaller and smaller germs could be repeated up to infinity, such a fractal dust would transform all the reactant *A* in the bulk instantly [43]. From a mathematical point of view this is not surprising, because any reactant *A* would have in its immediate vicinity a tiny catalytic germ, thus eliminating the diffusion step. However, such a behavior does not make sense from a practical point of view. The limitations of perfect reactions were recognized in 1949 by Collins and Kimball [31], who proposed replacing the Dirichlet boundary condition by the so-called Robin or radiative boundary condition on the catalytic surface:

(8) $$-D\frac{\partial [A](x,t)}{\partial n}=\kappa \;\left[A\right]\left(x,t\right).$$

This condition *postulates* that the (net) diffusive flux of reactant *A* coming onto the catalytic surface from the bulk (the left-hand side) is proportional to its concentration, [A], on that surface at each surface point. The proportionality coefficient κ, called the “reactivity” of the catalytic surface, can range from 0 for an inert surface to infinity for a perfectly reactive surface. In the former case, the diffusive flux of reactants is zero, meaning that no reaction occurs on that surface. In the latter case, the division by κ and the limit κ→∞ reduce Equation (8) back to the Dirichlet boundary condition [A](x,t)=0 on the surface of *C*. Note that the reactivity κ (in units m/s) can also be expressed in terms of a forward reaction constant $k_{\mathrm{on}}$ (in units m${}^{3}$/s/mol or 1/M/s) as $k_{\mathrm{on}}=\kappa N_{A}S_{C}$, where $N_{A}$ is the Avogadro number, and $S_{C}$ is the surface area of the catalytic surface. Figure 1 (bottom row) illustrates the effect of partial reactivity on the concentration of reactants near the catalytic sphere of radius *R*. The depletion zone is thinner and grows slower than in the case of perfect reactions. Moreover, the overall reaction rate J(t) is finite over a short time period: $J\left(0\right)=4\pi R^{2}\kappa {\left[A\right]}_{0}$. Indeed, only the molecules near the catalyst (of surface area $4\pi R^{2}$) can react at short timescales, and their contribution is now limited by chemical kinetics, i.e., by the time needed for chemical transformation (4), which is controlled by the reactivity κ. As time increases, molecules from more distant locations arrive onto the catalyst and can thus contribute. Over long timescales, the region near the catalyst is depleted, and reactant *A* needs to diffuse towards the catalyst from very distant locations. In this limit, one obtains $J\left(\infty \right)=4\pi RD{\left[A\right]}_{0}/\left(1+D/\left(\kappa R\right)\right)$, i.e., the overall production is, therefore, diffusion-limited. In other words, the overall production exhibits a transition from the reaction-limited regime over short timescales to the diffusion-limited regime over long timescales.

**Figure 2:**
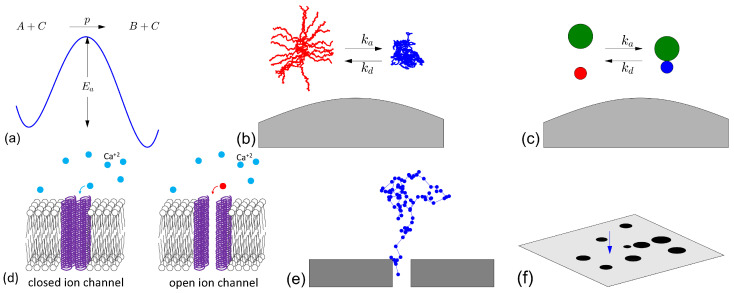
Various microscopic origins of imperfect surface reactions. (**a**) When the reactant *A* arrives onto the catalytic surface *C*, an activation energy barrier $E_{a}$ has to be overcome for a chemical transformation of *A* into *B*; if failed, the reactant leaves the vicinity of *C* and, thus, resumes its bulk diffusion. (**b**) A macromolecule can spontaneously switch its conformational state from “active” (in red) to “passive” (in blue) with the rate $k_{a}$, and back (with the rate $k_{d}$), while its reaction on the catalytic surface (in gray) or with another macromolecule (a receptor, an enzyme, a DNA strand, etc.) is only possible in the “active” conformational state. (**c**) The reactant can be temporarily trapped by a buffer molecule (in green) that makes it inactive for the considered surface reaction; their association/dissociation kinetics is usually described by forward and backward rates $k_{a}$ and $k_{d}$. (**d**) An ion can pass through an open channel, while it is reflected back from a closed channel. (**e**) The escape of a semi-flexible polymer through a small hole can be described by an entropic barrier that leads to partial reactivity when the first arrival to the hole does not guarantee the passage. (**f**) An inert (gray) surface is covered by reactive catalytic germs (black spots) so that the reactant may fail to react upon the first arrival, and thus, resumes its bulk diffusion until the next encounter, and so on. Similarly, a protein can search for a specific (target) site on a DNA chain for successful binding.

The partial reactivity of the surface, described by the Robin boundary condition (8), can model various microscopic mechanisms of imperfect reactions [34,44], as illustrated in Figure 2. In physical chemistry, once the reactant *A* arrives onto the catalytic surface, it has to overcome an activation energy barrier in order to react [45,46]. This activation energy determines the probability *p* of the reaction attempt being successful. However, the reactant may fail its reaction attempt (with probability 1−p) by leaving the proximity of the catalytic surface and, thus, resuming its diffusion until the next encounter, and so on. In this setting, the microscopic interaction determines the probability *p*, which, in turn, fixes the effective macroscopic reactivity $\kappa =\frac{D}{a}\;\frac{p}{1-p}$, where *a* is the width of the reactive layer near the catalytic surface (i.e., the interaction range, which is typically of the order of a nanometer) [47]. Varying *p* from 0 to 1 covers the whole range of reactivities from 0 to +∞. In the biochemical context, conformational changes of a macromolecule between nearly isoenergetic folded states can alter its function; this mechanism is primarily important for protein–ligand and protein–protein recognition [48,49,50]. When such a protein arrives onto the catalytic surface (its reaction partner), it has to be in an appropriate conformational state (with probability *p*) to be able to initiate the reaction (4); otherwise, the protein leaves the catalytic surface and restarts its bulk diffusion [51]. Even small particles such as calcium ions can spontaneously lose their reactivity via reversible binding to buffer molecules. This is the basis of one of the regulatory mechanisms in neuron signaling, where tuning the concentration of buffer molecules inside a presynaptic bouton controls the ability of calcium ions to reach calcium-sensing proteins that trigger the vesicular release of neurotransmitters (see [52] and references therein). In the microcellular context, the catalytic surface may represent a plasma membrane of a cell or of a nucleus, while the reaction event may consist in the passage through a channel on that membrane; such a “reaction” occurs if the channel is open (with probability *p*), while the reactant is reflected back from a closed channel [53,54,55]. Even if the channel is always open (e.g., just a hole in a container or in a filter), there is an entropic barrier that may prohibit the escape from the confining domain and lead to reflection and resumed diffusion [56,57,58]. In heterogeneous catalysis, the macroscopic reactivity κ may account for micro-heterogeneity of the catalytic surface, which is not fully covered by catalytic germs; in this case, *p* is the probability of hitting the catalytic surface at the catalytic germ (and thus of reacting), while 1−p is the probability of arriving at the inert part of the surface and, thus, being reflected. The homogenization of spatially heterogeneous catalytic surfaces leads to the Robin boundary condition (8), in which the reactivity κ effectively accounts for distributed reactive spots [59,60,61,62,63,64]. For instance, in the seminal work by Berg and Purcell [59], the probability *p* was found for a spherical cell of radius *R* covered by *N* disk-shaped receptors of radius *a*: p=Na/(Na+πR).

The partial reactivity adds an important intermediate step to diffusion-controlled reactions: after the first arrival onto the catalytic surface, the reactant executes a sequence of diffusive explorations of the bulk near the catalytic surface after each failed reaction attempt. This step may considerably slow up the overall production, while the shape and reactivity of the catalytic surface are linked through diffusion in a sophisticated way. Note that the same problem emerges in the context of semi-permeable membranes in biology and blocking electrodes in electrochemistry [65,66,67]. The role of reactivity (or, equivalently, permeability or resistivity) on the overall production has been thoroughly investigated [17,20,68,69,70,71]. For instance, B. Sapoval and co-workers discussed the role of the “reaction length” D/κ as a physical scale for oxygen capture efficiency in human lungs [29].

## 4. Various Extensions

The basic description of diffusion-controlled reactions via Equations (5) and (8) has been generalized in different ways. Most efforts were dedicated to extensions of the diffusion Equation (5) that describes the simplest diffusive motion of reactants, the so-called Brownian motion. For instance, the Fokker–Planck equation allows one to incorporate the effects of external potentials (e.g., an electric field acting on a charged particle), anisotropy, and space- and/or time-dependence of the diffusion coefficient [72,73,74,75]. Fractional space and time derivatives can further include nonlocal displacements and memory effects in continuous-time random walks [76,77,78,79]. Diffusing diffusivity and switching diffusivity models were proposed to describe the diffusive transport in dynamically heterogeneous media or in the presence of buffer molecules that may reversibly bind the reactant and, thus, randomly change its diffusion coefficient [80,81,82,83]. The addition of a linear term proportional to [A] on the right-hand side of the diffusion Equation (5) can account for first-order disintegration mechanisms such as photo-bleaching, bulk relaxation, radioactive decay, or a finite lifetime of the reactant [84,85,86], as well as the effect of diffusion-sensitizing magnetic-field-gradient encoding in diffusion magnetic resonance imaging [21]. Moreover, the diffusion equation with nonlinear terms in [A] can describe reaction waves and many out-of-equilibrium chemical reactions involving “activators” and “inhibitors” (e.g., Belousov–Zhabotinsky reaction), paving a way to the theory of pattern formations initiated by A. Turing [1,87].

The above extensions generally employ the canonical Dirichlet or Robin boundary conditions. Such a “persistence” can partly be explained by two mathematical reasons: (i) the Laplace operator with either of these boundary conditions is known to be self-adjoint (Hermitian), allowing one to rely on powerful methods of spectral theory and to borrow numerous tools from quantum mechanics; (ii) the diffusion equation with these boundary conditions has a straightforward probabilistic interpretation that provides more intuitive insights onto the studied diffusion reaction processes, offers efficient Monte Carlo simulations, and helps to extend the macroscopic description in terms of concentrations to single-molecule experiments. In fact, many biochemical reactions involve proteins that are not abundant inside living cells. When the number of proteins is relatively small (e.g., a few tens or a few hundred transcription factors in a bacterium [88]), the macroscopic notion of concentration may be inapplicable, and the overall reaction rate may be uninformative or even misleading, while *fluctuations* become critically important. Such reactions require, therefore, a probabilistic description in terms of the survival probability of a single reactant molecule and the probability density of the first-reaction time [89,90]. In many settings, the survival probability of a single molecule obeys the same Equations, (5) and (8), and hence is equal to the rescaled concentration $\left[A\right]\left(x,t\right)/{\left[A\right]}_{0}$. This equivalence bridges the macroscopic and probabilistic descriptions, providing complementary insights and opening efficient ways to analyze and interpret single-molecule experiments [91,92,93,94,95,96,97,98,99,100,101].

At the same time, the Robin boundary condition (8) remains limited to modeling rather simple surface reactions with a *constant* reactivity. Consideration of time- and/or space-dependent reactivity is one natural extension (see [102] and references therein). Another important extension concerns reversible reactions such as binding/unbinding, association/dissociation, and adsorption/desorption kinetics, in which the reactant can be temporarily bound to the surface (or to another molecule). The exchange between free particles and those bound on the surface can be incorporated through the “back-reaction” boundary condition, also known as “generalized radiation” or the “generalized Collins–Kimball” boundary condition [103,104,105,106,107,108,109,110,111]. Application of the Laplace transform with respect to time, $\tilde{\left[A\right]}\left(x,s\right)=\int_{0}^{\infty }dt\;e^{-st}\;\left[A\right]\left(x,t\right)$, reduces this boundary condition to the Robin boundary condition (8) with *s*-dependent reactivity κ(s) (see details in [111]). In this way, reversible and irreversible diffusion-controlled reactions admit essentially the same mathematical description in the Laplace domain (in terms of *s*); in turn, the *s*-dependent reactivity results in fundamentally different behaviors in the time domain (in terms of *t*). In addition, one can further relax the assumption of an immobile bound state and allow for diffusion on the surface. The efficiency of such intermittent search dynamics with alternating phases of bulk and surface diffusion has been thoroughly investigated [112,113,114,115,116,117,118,119,120,121] (see also a review in [122]).

## 5. Beyond the Conventional Framework

To handle more general surface reaction mechanisms such as, e.g., deactivation or passivation of catalysts [123,124], or progressive activation of enzymes, an alternative theoretical description of diffusion-controlled reactions was proposed [125]. This so-called encounter-based approach originates from the theory of reflected stochastic processes in confined domains and relies on the concept of the boundary local time *ℓ*—a rescaled number of encounters between the reactant and the catalytic surface. In this approach, one can *disentangle* the respective roles of the shape and reactivity of the catalytic surface. In fact, the concentration of reactant *A* can be represented as

(9) $$\left[A\right]\left(x,t\right)=\int_{0}^{\infty }d\ell \;e^{-\ell \kappa /D}\;\rho \left(\ell ,x,t\right),$$

where ρ(ℓ,x,t) describes the statistics of encounters with an inert surface. In other words, the function ρ(ℓ,x,t) encodes how the shape of the catalytic surface affects the diffusive dynamics, whereas the exponential factor $e^{-\ell \kappa /D}$ incorporates the reactivity κ that was *implicitly* imposed via the Robin boundary condition (8) in the conventional approach. As the successful surface reaction is preceded by a sequence of failed reaction attempts at each encounter, the exponential factor in Equation (9) can be interpreted as the exponential probability law, $P\left\{a\hat{n}>\ell \right\}=e^{-\ell \kappa /D}$, for the random number $\hat{n}$ of encounters in that sequence. Due to the self-similar nature of Brownian motion, the number of encounters has to be rescaled by the width *a* of a thin surface layer, in which the molecule can interact with the catalytic surface (see details in [125]). While the statistics of encounters has been investigated for simple confinements [126,127,128,129,130,131,132], its shape dependence for porous media representing industrial catalysts or biological environments remains still unknown.

Most importantly, one can replace the exponential factor in Equation (9), which incorporated the effect of a constant reactivity κ, by another probability law $P\left\{a\hat{n}>\ell \right\}=\Psi \left(\ell \right)$, to model more sophisticated surface reaction mechanisms with an encounter-dependent reactivity,

(10) $$\kappa \left(\ell \right)=D\frac{-\frac{d}{d\ell }\Psi \left(\ell \right)}{\Psi (\ell )}\;.$$

**Figure 3:**
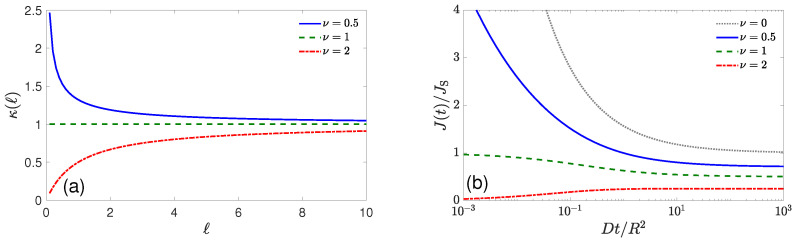
(**a**) Encounter-dependent reactivity κ(ℓ) from the gamma model with q=1 and three values of ν. (**b**) The overall reaction rate J(t) on a spherical catalyst of radius *R*, rescaled by Smoluchowski’s rate $J_{S}=4\pi DR{\left[A\right]}_{0}$, with q=1 and three values of ν. Dotted curve represents Equation (7) for a perfectly reactive sphere (it formally corresponds to ν=0).

If $\Psi \left(\ell \right)=e^{-\ell \kappa /D}$, this formula yields the constant reactivity considered above, κ(ℓ)=κ, and ensures the Markovian character of the binding reaction. However, another *choice* of the function Ψ(ℓ) allows one to implement the reactivity of the catalytic surface that depends on how many times the reactant has encountered it. To illustrate this idea, let us consider the gamma model, by choosing Ψ(ℓ)=Γ(ν,qℓ)/Γ(ν,0), where q>0 and ν>0 are two parameters, and $\Gamma \left(\nu ,z\right)=\int_{z}^{\infty }dx\;x^{\nu -1}e^{-x}$ is the upper incomplete gamma function. For ν=1, one has $\Gamma \left(1,z\right)=e^{-z}$, thus retrieving the above setting of constant reactivity κ=qD. Figure 3 illustrates the corresponding encounter-dependent reactivity κ(ℓ) given by Equation (10) (panel a), and the overall reaction rate J(t) on a spherical catalyst of radius *R* (panel b) that can be found in the framework of the encounter-based approach [125]. When 0<ν<1, the catalytic surface is highly reactive at the beginning and then reaches a constant reactivity qD. This situation can model a progressive passivation of the catalytic surface by repeated encounters with the reactant, up to a constant level. As expected, the diffusive flux is high on short timescales and then decreases to a constant steady-state level. Note that ν=0 formally corresponds to a perfect reaction, with Smoluchowski’s rate given in Equation (7). The particular value ν=1 yields the constant reactivity, independent of the number of encounters, for which the diffusive flux is constant on short timescales, $4\pi R^{2}qD{\left[A\right]}_{0}$, and slowly decreases to another constant on long timescales, as predicted by Collins and Kimball [31]. In turn, if ν>1, the catalytic surface is passive at the beginning and then reaches a constant reactivity. This situation can model progressive activation of that catalytic surface. Accordingly, the overall reaction rate is zero on short timescales and then increases to a constant steady-state level. Choosing an appropriate function Ψ(ℓ), one can produce the desired shape of the encounter-dependent reactivity κ(ℓ) that opens a way to model various surface reaction mechanisms.

The encounter-based approach goes far beyond the conventional theory of diffusion-controlled reactions described by the Dirichlet or Robin boundary conditions. From the mathematical point of view, the description of a general surface reaction with an encounter-dependent reactivity κ(ℓ) is not reducible to the Robin boundary condition. As a consequence, Laplacian eigenfunctions, that are conventionally used in spectral expansions, need to be replaced by so-called Steklov eigenfunctions [125,133]. Though being less known in the context of chemical reactions, these eigenfunctions turn out to be particularly well suited for describing diffusive explorations near a catalytic surface between successive reaction attempts. Several extensions of the encounter-based approach have already been explored, such as (i) the inclusion of an external potential that leads to a biased or drifted motion [134]; (ii) the effects of stochastic resetting [135,136] of the position and of the boundary local time on diffusion-controlled reactions [137,138]; (iii) the cooperative search by multiple independent particles and the related extreme first-passage statistics [139]; (iv) the escape problem [140]; and (v) non-Markovian binding/unbinding kinetics [111]. Moreover, the same concepts can be applied to describe diffusive permeation across membranes [141,142,143]. Despite these recent advances, there are many open questions and promising perspectives for future developments, such as merging anomalous bulk diffusions with generalized surface reactions, the effect of sophisticated geometric confinements on the encounter statistics, the competition of multiple reactive centers for capturing a limited amount of diffusing reactants, indirect coupling of different reactants through encounter-dependent catalytic surfaces, and inference of appropriate surface reaction models from experimental data, to name but a few.

## 6. Conclusions

In summary, we have reviewed the major steps in the long history of developments in the theory of diffusion-controlled reactions. M. von Smoluchowski first recognized the importance of the diffusion step, during which the reactants have to meet each other. He also put forward the diffusion equation to describe the dynamics of reactants in the bulk and boundary conditions to account for the reaction on the surface. His mechanism of perfect reactions upon the first encounter was then improved by Collins and Kimball. While most later theoretical efforts were dedicated to improvements of the bulk dynamics, an encounter-based approach was recently developed to enable more general surface reaction mechanisms. This approach has already shown many advantages such as probabilistic insights into surface reactions, disentanglement of the impacts of shape and reactivity of the catalytic surface, flexibility in characterization of diffusive explorations near the reactive surface, etc. In particular, the concept of encounter-dependent reactivity allows one to describe an action of reactants on the catalytic surface, and such a feedback may potentially be relevant in various biochemical and electrochemical settings. There are still many open questions and current developments, aiming to understand the mathematical formalism of the encounter-based approach, relating the shape of the catalytic surface to the spectral properties of the underlying operators, elaborating various extensions, and uncovering potential applications in chemistry and biochemistry.

## Data Availability

No new data were created or analyzed in this study. Data are contained within the article.
